# Trihexyphenidyl Ameliorates Depression-like Behaviors in Adult Zebrafish Exposed to Chronic Unpredictable Stress, Consistent with Regulation of the MAPK Signaling Pathway

**DOI:** 10.3390/biom16050678

**Published:** 2026-05-02

**Authors:** Siqi Hu, Yedong Yao, Siyuan Li, Leqing Zhan, Rihua Feng, Dongting Zhangsun, Sulan Luo, Xiaopeng Zhu

**Affiliations:** Guangxi Key Laboratory of Special Biomedicine, School of Medicine, Guangxi University, Nanning 530004, China; 2328302007@st.gxu.edu.cn (S.H.); 2237030128@st.gxu.edu.cn (Y.Y.); 2328403002@st.gxu.edu.cn (S.L.); 2437030209@st.gxu.edu.cn (L.Z.); 2222310350@st.gxu.edu.cn (R.F.); zhangsundt@163.com (D.Z.)

**Keywords:** chronic unpredictable stress, zebrafish, trihexyphenidyl, MAPK signaling pathway, neuroinflammation

## Abstract

Depression is a complex mental and neurological disorder and has become one of the most serious public health issues in modern society. Trihexyphenidyl (THY) is a traditional drug used to treat Parkinson’s disease. Recent studies have suggested that it may play a role in regulating neurotransmitters and protecting neurons, but its potential for treating depression has not been fully explored, and how it works remains unclear. Therefore, we examined the effects of THY on depression-like behaviors in zebrafish caused by chronic unpredictable stress (CUS). Our results showed that THY significantly attenuated the CUS-induced decrease in exploratory behavior and shortened the CUS-induced increase in latency time. At the tissue level, THY effectively attenuated the thinning of the optic tectum and the loss of Nissl bodies caused by CUS. In addition, THY reversed the CUS-induced increase in stress hormone levels and reduction in neurotransmitter content. Through network pharmacology and transcriptome sequencing analysis, we found that the mechanisms underlying depression-like behaviors and the antidepressant effects of THY might be related to the MAPK signaling pathway. Further experiments showed that THY regulated the CUS-induced activation of the MAPK signaling pathway, improved the abnormal activation of microglia and damage to astrocytes, and reduced the expression of pro-inflammatory factors, thereby easing neuroinflammation and improving depression-like behaviors. In summary, this study explored the potential mechanism of THY ameliorating depressive-like behaviors and provided basic theoretical evidence.

## 1. Introduction

Depression is a serious mental disorder that significantly harms physical and mental health. Its prevalence continues to rise worldwide, making it a major public health concern [1]. The clinical symptoms of depression are characterized by a persistent low mood that is disproportionate to the patient’s circumstances, accompanied by loss of interest, slowed thinking, and abnormal appetite. In severe cases, this condition may lead to self-harm and suicidal behavior [2,3]. Behind these symptoms lie complex physiological and pathological changes. Long-term depression can cause overactivity of the hypothalamic–pituitary–adrenal (HPA) axis and imbalances in neurotransmitters in the brain, such as serotonin, dopamine (DA), and norepinephrine (NE) [4]. Additionally, it can trigger abnormal activation of microglia and damage to astrocytes [5,6], leading to neuroinflammation, which harms neurons and disrupts their normal structure and function [7,8].

Research indicates that depression is associated with dysfunction in multiple signaling pathways. Among these, the mitogen-activated protein kinase (MAPK) signaling pathway plays a critical role in its pathogenesis [9,10]. Widely distributed throughout the central nervous system, the MAPK pathway is involved in various biological processes such as inflammation, cell cycle regulation, and cell death, which are closely linked to the development of depression [11,12]. Evidence suggests that the modulating aberrant MAPK activation can alleviate depressive-like behaviors in experimental animals [13]. At the same time, depression shares complex and common regulatory mechanisms with many chronic diseases, such as Parkinson’s disease and bipolar disorder [14].

In light of the above, we have focused on trihexyphenidyl (THY), a traditional medication used to treat Parkinson’s disease. Its mechanism of action involves selectively blocking cholinergic pathways in the striatum, with minimal peripheral effects, thereby helping to restore the balance of DA and acetylcholine in the brains of Parkinson’s patients and improve their symptoms; however, its exact mechanism of action has not yet been fully elucidated [15]. Currently, due to abuse and severe adverse reactions (such as nausea, vomiting, urinary retention), THY is not considered a first-line treatment for Parkinson’s disease [16]. Recent studies have revealed that it possesses neuroprotective and antioxidant properties [17]. In a human study, Katsumasa Sogo et al. reported its potential efficacy in reducing flashbacks in patients with post-traumatic stress disorder [18]. Research by Zeina A. Althanoon and colleagues showed that THY can induce psychostimulant-like locomotor excitation by enhancing dopamine transmission and producing antidepressant-like effects [19]. Furthermore, Anthony M. Downs et al. found that THY restored dopamine neurotransmission deficits in an early-onset isolated dystonia mouse model [20]. Considering these multifaceted neuroregulatory effects and the shared pathogenic mechanisms between depression and other psychiatric disorders, we speculate that THY may have therapeutic potential for depression.

Meanwhile, the zebrafish has emerged as a valuable vertebrate model organism, sharing over 70% genetic homology with humans and possessing a central nervous system and social behaviors highly comparable to those of humans [21]. Zebrafish have been employed to model both acute and chronic stress, facilitating their widespread use in studies on depression, anxiety, post-traumatic stress disorder, and related emotional disorders [22,23,24]. As a complement to mammalian animal models and in vitro experiments, zebrafish can be used to construct induced depression models and further explore the mechanisms underlying depression.

In this experiment, we employed a zebrafish chronic unpredictable stress (CUS) model to explore whether THY can ameliorate depression-like behaviors and to explore its underlying molecular mechanisms of antidepressant action.

## 2. Materials and Methods

### 2.1. Main Materials

THY (CAS: 52-49-3) was obtained from Shanghai Macklin Biochemical Co., Ltd. (Shanghai, China). The following primary antibodies were used: Iba 1 Antibody (Cat# T57217), GFAP Antibody (Cat# T55424), p38 MAPK (Cat# T55600), JNK1/2/3 Antibody (Cat# PA7042), β-actin (Cat# P30002), and Goat Anti-Rabbit Mouse IgG-HRP (Cat# M21003). These were purchased from Abmart Inc. (Shanghai, China). Phospho-p38 MAPK (P-p38, Cat# F0159), Phospho-JNK1/2/3 (P-JNK, Cat# F1572) provided by Selleck (Shanghai, China). ChamQ Universal SYBR qPCR Master Mix (Cat# Q711-02) and HiScript III RT SuperMix for qPCR (+gDNA wiper) (Cat# R323-01) were purchased from Vazyme Biotech (Nanjing, China). An enhanced chemiluminescent (ECL) plus reagent kit was bought from Biosharp (Cat# BL523B, Beijing, China).

### 2.2. Experimental Animals and Rearing Environment

In this study, wild-type adult zebrafish (*Danio rerio*) (AB strain, 5–7 months old) were purchased from a commercial supplier (Shanghai Yinuo Aquarium Technology Co., Ltd., Shanghai, China). All experimental animals were acclimated to the feeding environment for two weeks before the experiment. The stocking density was two fish per liter of water, and an aerator was used to increase dissolved oxygen levels to meet their biological oxygen demand. The fish were fed brine shrimp three times a day. The water temperature was set at 28 ± 1 °C, the pH was maintained at 7.3–7.4, and the light cycle was 14 h of light and 10 h of darkness (lights on at 8:00 a.m.). All animal procedures were approved by the Animal Ethics Committee of Guangxi University (GXU-2024-261) and were carried out in strict accordance with the guidelines established by this committee.

### 2.3. Model Establishment and Drug Treatment

After a two-week adaptation period, the fish were first divided into a Control group (non-stressed group) and a CUS group (stressed group). The CUS group all received 3-week experimental modeling based on the zebrafish CUS protocol proposed by Song et al. [25]. Behavioral tests and analyses were performed on zebrafish randomly selected from the Control and CUS groups to verify the successful establishment of the model (Figure S1). The CUS group was further divided into the Model group, low-dose THY group (50 μg/L) and high-dose THY group (250 μg/L) (randomly selected and assigned by a blinded researcher). This resulted in four final groups: Control group, Model group, low-dose THY group (50 μg/L), and high-dose THY group (250 μg/L), each with 20 fish. The volume of the breeding water was controlled at 10 L. The dosage was selected based on the maximum tolerated dose from a preliminary acute toxicity study (Table S1 and Figure S2). The water was replaced every 24 h, and the medication was administered simultaneously. The final drug concentrations in the breeding water were 50 μg/L and 250 μg/L. This process lasted for 7 days.

### 2.4. Behavioral Tests

To evaluate the effects of modeling and drug administration on zebrafish behavior, behavioral tests were performed on the zebrafish. The tests were conducted between 9 a.m. and 5 p.m., with the water temperature controlled at 28 ± 1 °C. Zebrafish behavior was analyzed in the order of the novel tank test (NTT), light–dark box test (LDB), and open field test (OFT), with only one test performed per day to ensure minimal inter-test effects.

The NTT setup was a glass tank (22 × 5 × 20, cm) virtually divided into top and bottom zones by a marked line. During the 5 min test, the following were measured: number of entries into the top zone, duration spent in the top zone, distance in the top zone, and freezing time in the bottom zone. The LDB test used a rectangular container (40 × 23 × 25, cm) divided into light and dark zones. Measurements recorded during the 5 min test included number of entries into the light zone and time spent in the light zone. The OFT was performed in a white square plastic arena (35 × 35 × 10, cm). Over the 15 min test period, we measured how many times the fish entered the central area and how long it stayed there. All behavioral tests were recorded by a trained blinded observer. Data analysis was independently performed by another researcher who was fully blinded to the experimental grouping, using the SMART 3.0 behavioral analysis system (Panlab, Spain) for small animals.

### 2.5. Hematoxylin and Eosin (H&E) Staining and Nissl Staining

After the zebrafish were sacrificed by ice-bath, the body weight and body length of each zebrafish were recorded (Figure S3). Three individual brains were collected from each group and prepared into paraffin sections. For H&E staining, the paraffin sections were baked at 70 °C for 1 h. The sections were then deparaffinized in a gradient of xylene and 100%, 90%, 80%, and 70% ethanol. They were stained with hematoxylin solution for 3 min, differentiated in differentiation solution for 3–5 s, blued in bluing solution for 3–6 s, and rinsed with distilled water. The sections were then stained with eosin solution for 15 s. After dehydration and clearing, the sections were mounted.

Similarly, for Nissl staining, the paraffin sections were deparaffinized, washed with distilled water for 5 min, incubated in Nissl stain for 1 h, and rinsed with running water until no color remained on the slides. The sections were dried at 65 °C, dehydrated, mounted, and observed under an inverted fluorescence microscope (Nikon Eclipse E100; Nikon, Tokyo, Japan). Histological quantification was performed by two trained researchers who were blinded to the experiment.

### 2.6. Detection of Stress Hormone and Neurotransmitter Levels

Place individual zebrafish into EP tubes, add 1 mL of phosphate-buffered saline (PBS) per fish, and add 350 μL of PBS to brain tissue samples. Six zebrafish were used in each group. The samples were homogenized using a homogenizer (KZ-III-F; Servicebio, Wuhan, China). After centrifugation at 12,000× *g* for 15 min at 4 °C, the supernatant was collected and combined as the test sample. The levels of corticotropin-releasing hormone (CRH), adrenocorticotropic hormone (ACTH), and cortisol (CORT) (in body), as well as DA, NE, and 5-hydroxytryptamine (5-HT) (in brain tissues) were measured using Enzyme-Linked Immunosorbent Assay (ELISA) kits (Shanghai Xinyu Biotech Co., Ltd., Shanghai, China) according to the manufacturer’s instructions.

### 2.7. Gene Expression Detection

Total mRNA was extracted from zebrafish brain tissue (5 brains per group). RNA was reverse transcribed using the HiScript III RT SuperMix for qPCR (+gDNA wiper) kit (Vazyme Biotech, Nanjing, China), and qRT-PCR was performed with the ChamQ Universal SYBR qPCR Master Mix(Vazyme Biotech, Nanjing, China). The amplification program was set as follows: pre-denaturation at 95 °C for 30 s; cycling parameters of 95 °C for 10 s and 55 °C for 20 s (annealing/extension), for a total of 48 cycles. Data analysis was conducted using the 2^−ΔΔCT^ relative quantification method. Primer sequences are listed in Table S2.

### 2.8. Immunofluorescence and Immunohistochemistry on Brain Tissue

For immunofluorescence, frozen tissue sections were placed at room temperature for 10 min to equilibrate and fixed with 4% paraformaldehyde for 30 min. The sections were permeabilized with PBS with tween-20 for 10 min and washed twice with PBS. After antigen retrieval, they were blocked with 5% bovine serum albumin for 1 h and then incubated with primary antibodies against ionized calcium-binding adaptor molecule 1 (Iba 1, 1:200) and glial fibrillary acidic protein (GFAP, 1:200) overnight at 4 °C. Subsequently, Cy3-conjugated secondary antibodies (1:200) were applied and incubated at room temperature for 2 h in the dark. Following PBS washes, DAPI solution (1:250) was added and incubated at room temperature for 10 min in the dark. Images were captured using an inverted fluorescence microscope, and the mean fluorescence intensity of each image was quantified using ImageJ 1.48s software.

Similarly, for immunohistochemistry, paraffin sections were baked at 70 °C for 2 h and subjected to gradient deparaffinization and rehydration using xylene and ethanol. After antigen retrieval, endogenous peroxidase activity was blocked with 3% H_2_O_2_. The sections were blocked with 5% bovine serum albumin and then incubated with primary antibodies against JNK (1:200) and p38 MAPK (1:200), overnight at 4 °C. Secondary antibodies were applied and incubated at room temperature for 2 h. Immunoreactivity was visualized using diaminobenzidine staining chromogen, and nuclei were counterstained with hematoxylin. After mounting, the sections were observed under an inverted fluorescence microscope.

### 2.9. Protein Expression Detection

Protein extraction was performed using radioimmunoprecipitation assay lysis buffer. Tissue samples were homogenized at 4 °C, then centrifuged at 12,000× *g* for 15 min at 4 °C, and the supernatant was collected. The protein concentration of the samples was quantified using the BCA Protein Quantification Kit (Vazyme Biotech, Nanjing, China). Lysates containing 30 μg of total protein were denatured in 5×SDS loading buffer (P0015; Beyotime Biotechnology, Shanghai, China) at 95 °C for 15 min. After separation by SDS-PAGE gel electrophoresis, they were transferred to a 0.45 μm polyvinylidene fluoride membrane (Millipore, Bedford, MA, USA). The membrane was blocked with 5% skimmed milk at room temperature for 2 h. Subsequently, the polyvinylidene fluoride membrane was incubated with primary antibodies overnight at 4 °C. After washing, the membrane was incubated with the secondary antibody Goat Anti-Rabbit/Mouse IgG-HRP (1:3000) for 2 h. During this step, the membrane was washed three times with tris-borate-sodium tween-20 buffer for 10 min each. Finally, the ECL chemiluminescence reagent was evenly applied to cover the polyvinylidene fluoride membrane, and images were captured using a chemiluminescence imaging system. The gray values of target protein bands were analyzed with ImageJ 1.48s software, and relative expression levels were calculated using β-actin as the internal reference.

### 2.10. Transcriptome

To explore the molecular mechanisms underlying depression-like behaviors induced by CUS in zebrafish, we performed transcriptomic analysis. The concentration, purity, and integrity of RNA extracted from the Control group and the Model group were detected to ensure that the extracted RNA met the requirements for subsequent sequencing. Enriched mRNA was fragmented, reverse transcribed into cDNA, and used to construct a cDNA library for sequencing. After the sequencing data was downloaded, bioinformatics analysis was conducted using the workflow provided by BMKCloud (www.biocloud.net). Filtered raw data yielded Clean Data, which was aligned against a designated reference genome to obtain Mapped Data. Using DESeq2_EBSeq software on BMKCloud, differentially expressed genes between samples were identified with the screening criteria: |lg_2_FC| ≥ 2 and FDR < 0.05. Gene Set Enrichment Analysis (GSEA) and Kyoto Encyclopedia of Genes and Genomes (KEGG) analyses were then performed on these differentially expressed genes.

### 2.11. Network Pharmacology

To predict the potential therapeutic targets and involved signaling pathways of THY, a network pharmacology analysis was performed on THY. The chemical structure of THY was retrieved from the PubChem database (https://pubchem.ncbi.nlm.nih.gov/). Eligible therapeutic targets (probability > 0) were screened using the Swiss Target Prediction (http://swisstargetprediction.ch, accessed on 3 August 2025) database and the PharmMapper (http://www.lilab-ecust.cn/pharmmapper/, accessed on 3 August 2025)) database. Genes associated with depression were collected and filtered from the GeneCards database (https://www.genecards.org, accessed on 4 August 2025), the OMIM database (https://omim.org, accessed on 5 August 2025) and the Therapeutic Target Database (https://db.idrblab.net, accessed on 6 August 2025), with the screening criterion of Relevance score ≥ 1 set for the GeneCards database.

Identify the potential active targets of THY for treating depression using Venny 2.1.0 (https://bioinfogp.cnb.csic.es/tools/venny/index.html, accessed on 6 August 2025). Select these targets and import them into the Search Tool for the Retrieval of Interacting Genes/Proteins database (https://string-db.org, accessed on 6 August 2025) for analysis. Export the analysis results to Cytoscape 3.10.3 software, and use the CytoHubba plugin to screen the protein–protein interaction (PPI) network. Based on the analytical data, select the top 20 key targets. Perform Gene Ontology (GO) functional annotation and KEGG pathway analysis on the obtained intersection targets using the Database for Annotation, Visualization and Integrated Discovery (DAVID) database (https://davidbioinformatics.nih.gov, accessed on 10 August 2025), and visualize the GO and KEGG pathway enrichment results via the Micro Bioinformatics Platform (http://www.Bioinformatics.com.cn/). Integrate the data of the top 20 KEGG pathways and their associated targets, then import the integrated data into Cytoscape 3.10.3 to construct an “active component–target–disease–pathway” network.

### 2.12. Molecular Docking

The 3D structure files of JNK (PDB ID: 4QTD) and p38 (PDB ID: 6ZWP) proteins were downloaded from the PDB database (https://www.rcsb.org). The protein structures were checked using PyMOL 2.3.0 software in preparation for docking. The 3D structure file of the small molecule ligand, THY, was downloaded from the PubChem database. The structure of the small molecule was then optimized using the MMFF94 force field in OpenBabel 3.1.1 software to ultimately obtain the optimal molecular structure in its lowest energy state.

Protein and ligand structures were prepared using AutoDock Tools 1.5.6: hydrogen atoms were added, rotatable bonds were defined for the small molecule, and files were saved in pdbqt format. Docking grids were centered on the co-crystallized ligand of each protein with the following box dimensions: for JNK, center (16, 15, 28) and size (53 × 62 × 79); for p38, center (0, 7, −17) and size (23 × 27 × 29). Semi-flexible docking was performed using AutoDock Vina 1.2.5 with an exhaustiveness of 25 and the Lamarckian genetic algorithm. The binding free energies and docking poses were recorded.

### 2.13. Statistical Analysis

Statistical analysis was performed using GraphPad Prism 9.5.1. All data are presented as mean ± standard error of the mean (mean ± SEM). After verifying the normality and homogeneity of variance of the data, one-way ANOVA was performed, and Dunnett’s test was used for multiple comparisons to assess differences from the control group. Otherwise, the non-parametric Kruskal–Wallis test was adopted, followed by Dunnett’s test for multiple comparisons. All experiments were repeated no fewer than three times. A value of *p* < 0.05 was considered statistically significant.

## 3. Results

### 3.1. THY Alleviates Depressive-like Behaviors in Zebrafish Induced by CUS

**Figure 1:**
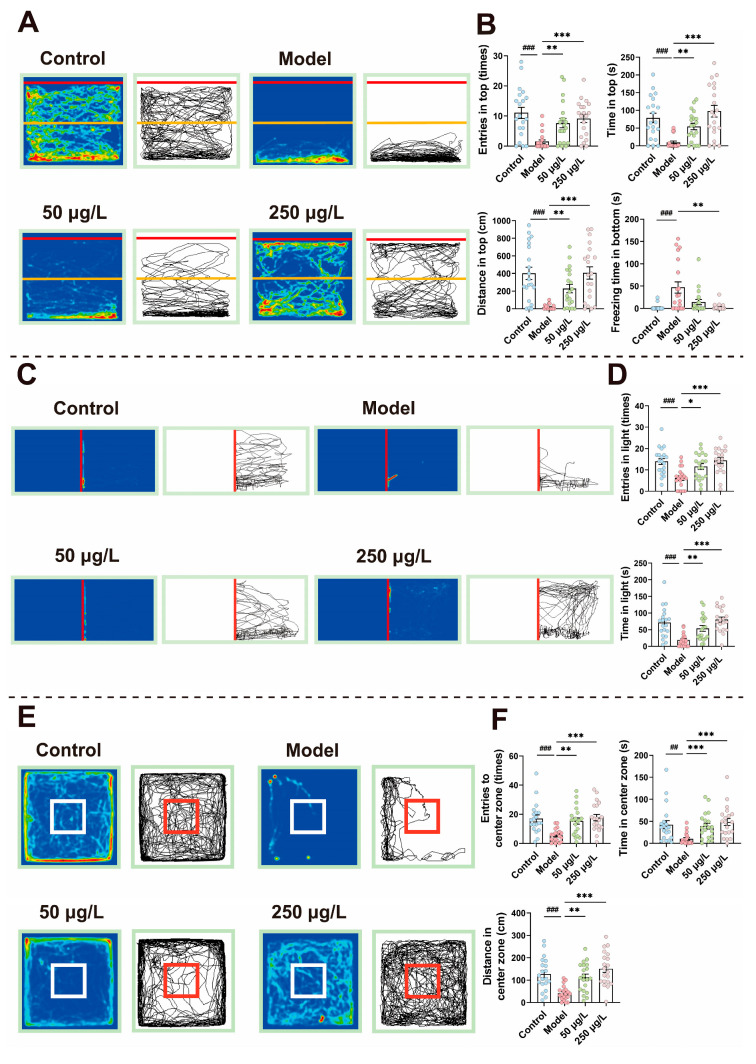
THY ameliorates depression-like behaviors induced by CUS. (**A**) Heatmaps and track plots of zebrafish exploration in the NTT (The red line represents the water surface and the yellow line divides the tank into the top and bottom sections). (**B**) Entries in top (times) (*H* = 23.2, *p* < 0.001), time in top (s) (*H* = 25.5, *p* < 0.001), distance in top (cm) (*H* = 28.9, *p* < 0.001) and freezing time in bottom (s) (*H* = 22.9, *p* < 0.001) in the NTT. (**C**) Heatmaps and track plots of zebrafish exploration in LDB (The area on the right of the red line is the bright zone, which is the area for statistical analysis). (**D**) Entries in light (times) (*F* = 8.61, *p* < 0.001) and time in light (s) (*F* = 28.5, *p* < 0.001) in the LDB. (**E**) Heatmaps and track plots of zebrafish exploration in the OFT (The red box represents the area for statistical analysis). (**F**) Entries to center zone (times) (*F* = 8.18, *p* < 0.001), time in center zone (s) (*H* = 22.5, *p* < 0.001), and distance in center zone (cm) (*H* = 25.9, *p* < 0.001) in the OFT. All data are presented as mean ± SEM. One-way ANOVA was used for overall group comparison, per group 20 fish. Compared to control group, ^##^ *p* < 0.01, ^###^ *p* < 0.001; Compared to model group, * *p* < 0.05, ** *p* < 0.01, *** *p* < 0.001.

As shown in Figure 1, in the NTT, the Model group showed significant reductions in the frequency of exploration in the upper zone (1.5 ± 0.62 times vs. 11.1 ± 1.86 times in controls, 86% decrease) (*H* = 23.2, *p* < 0.001), time spent in the upper zone (9.4 ± 3.9 s vs. 78.6 ± 13.3 s in controls, 88% decrease) (*H* = 25.5, *p* < 0.001), and distance travelled in the upper zone (17.2 ± 6.4 cm vs. 403.0 ± 70.6 cm in controls, 96% decrease) (*H* = 28.9, *p* < 0.001). In addition, freezing time at the bottom was significantly prolonged (47.2 ± 12.7 s vs. 2.1 ± 1.4 s in controls, 2148% increase) (*H* = 22.9, *p* < 0.001) (Figure 1A,B). These results indicate increased bottom-dwelling behavior and reduced exploratory motivation in CUS-exposed zebrafish. In the LDB, the Model group exhibited a significant decrease in the number of entries into the light zone (6.2 ± 1.1 times vs. 14.0 ± 1.4 times in controls, 56% decrease) (*F* = 8.61, *p* < 0.001) and time spent in the light zone (19.0 ± 4.1 s vs. 71.2 ± 10.2 s in controls, 73% decrease) (*F* = 28.5, *p* < 0.001) (Figure 1C,D), suggesting that CUS enhanced risk avoidance instinct. In the OFT, the Model group tended to stay in the periphery of the arena, showing significantly reduced exploration in the central zone: decreased number of entries into the central area (5.5 ± 0.9 times vs. 17.2 ± 2.4 times in controls, 68% decrease) (*F* = 8.18, *p* < 0.001), reduced time spent in the central area (10.3 ± 2.9 s vs. 42.6 ± 9.2 s in controls, 76% decrease) (*H* = 22.5, *p* < 0.001), and shortened distance travelled in the central area (41.7 ± 7.2 cm vs. 127.0 ± 16.2 cm in controls, 67% decrease) (*H* = 25.9, *p* < 0.001) (Figure 1E,F). Compared to the Control group, the Model group displayed stronger thigmotaxis and anxiety-like behavior. Following THY treatment, all the above depressive-like behaviors were reversed. Exploration activities in the LDB, NTT, and OFT were significantly enhanced, reflected by increased entries into the light, upper, and central zones, prolonged duration in these zones, and reduced freezing time, in a dose-dependent manner. These results indicate that THY effectively alleviates CUS-induced anxiety and depression-like behaviors in zebrafish.

### 3.2. THY Ameliorates CUS-Induced Neuronal Damage

**Figure 2:**
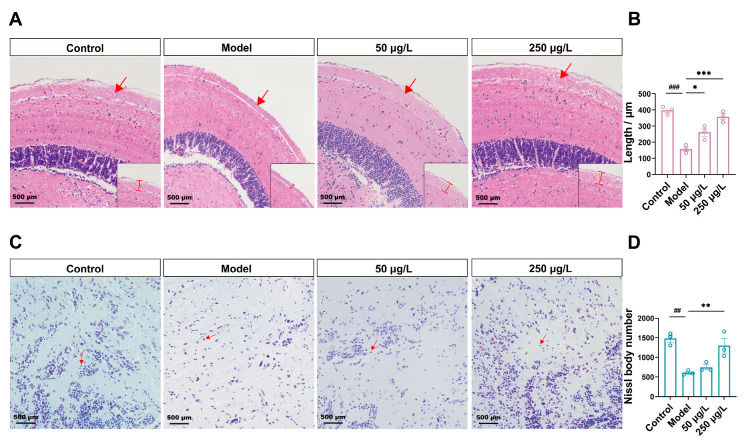
THY exerts a protective effect against neuronal injury induced by CUS. (**A**) H&E staining was used to evaluate the degree of neuronal damage and the thickness of the optic tectum (optic tectum indicated by the arrow). Scale bar, 500 µm. (**B**) Statistics of optic tectum thickness (*n* = 3) (*F* = 32.7, *p* < 0.001). (**C**) Nissl staining was utilized to detect the damage of neurons (Nissl bodies indicated by the arrow). Scale bar, 500 µm. (**D**) Statistics on the number of Nissl bodies (*n* = 3) (*F* = 14.6, *p* = 0.001). All data are presented as mean ± SEM. One-way ANOVA was used for overall group comparison. Compared to control group, ^##^ *p* < 0.01, ^###^ *p* < 0.001; Compared to model group, * *p* < 0.05, ** *p* < 0.01, *** *p* < 0.001.

Depression is often accompanied by neuronal damage and alterations in synaptic plasticity. As shown in Figure 2, H&E staining revealed that CUS exposure resulted in a significant reduction (by 61%) (*F* = 32.7, *p* < 0.001) in the thickness of the optic tectum in the Model group (Figure 2A,B). Moreover, the neuronal structure appeared damaged, with loosely arranged cells and expanded intercellular spaces. Nissl staining results revealed that Nissl bodies in the brains of the Control group zebrafish were neatly arranged and clearly visible. Compared with the Control group, the Model group showed a reduction in the number of Nissl bodies (by 59%) (*F* = 14.6, *p* = 0.001) and lighter staining (Figure 2C,D). After treatment with THY, the thickness of the optic tectum increased, neurons in the gray matter became more densely packed, and the number of Nissl bodies increased, resulting in darker staining. These findings indicate that THY attenuates the extent of neuronal damage in the zebrafish brain induced by CUS.

### 3.3. THY Modulated CUS-Induced Hyperactivation of the HPA Axis and Alterations in Neurotransmitter Levels

**Figure 3:**
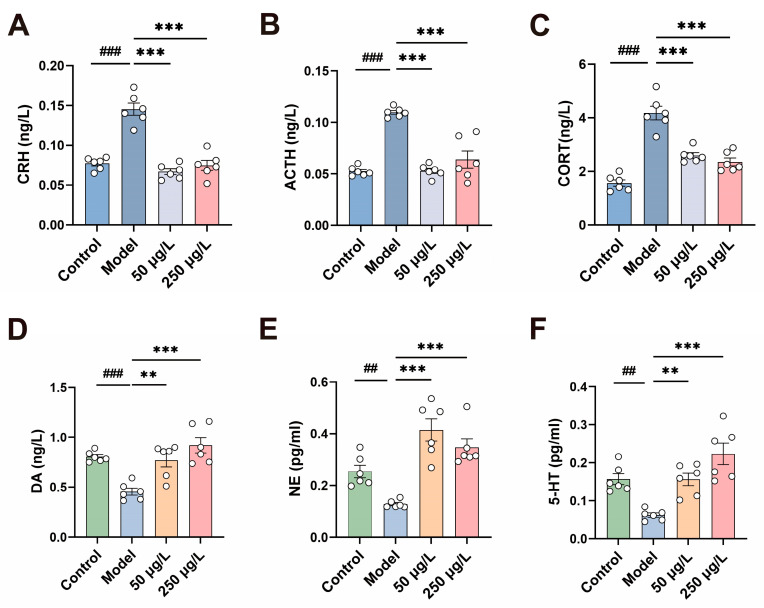
THY regulates stress hormones and neurotransmitters. (**A**–**C**) The levels of CRH (*F* = 43.4, *p* < 0.001), ACTH (*F* = 35.5, *p* < 0.001) and CORT (*F* = 42.9, *p* < 0.001) were measured by ELISA (*n* = 6). (**D**–**F**) The levels of DA (*F* = 13.2, *p* < 0.001), NE (*F* = 17.6, *p* < 0.001) and 5-HT (*F* = 13.5, *p* < 0.001) in zebrafish brains were measured by ELISA (*n* = 6). All data are presented as mean ± SEM. One-way ANOVA was used for overall group comparison. Compared to control group, ^##^ *p* < 0.01, ^###^ *p* < 0.001; Compared to model group, ** *p* < 0.01, *** *p* < 0.001.

Studies have shown that patients with depression frequently exhibit hyperactivity of the HPA axis. Excessive activation of the HPA axis leads to dysregulated secretion of stress hormones, thereby disrupting neuroendocrine homeostasis. The levels of CRH, ACTH, and CORT are key indicators reflecting HPA axis activity and are typically characteristically elevated in patients with depression. To verify the effects of THY on HPA axis function, ELISA was used in this study to detect the levels of the above hormones in zebrafish. As shown in Figure 3, compared with the Control group, the Model group exhibited significant increases in CRH (*F* = 43.4, *p* < 0.001), ACTH (*F* = 35.5, *p* < 0.001) and CORT (*F* = 42.9, *p* < 0.001) levels, with elevations of 0.9-fold, 1.1-fold, and 1.7-fold, respectively. However, THY treatment significantly inhibited the release of these hormones (Figure 3A–C).

In addition, the levels of monoamine neurotransmitters in the zebrafish brain were measured. Long-term high levels of cortisol disturb neurotransmitter homeostasis, reduce the synthesis of monoamine neurotransmitters, and impair neural transmission. Following CUS induction, the levels of DA (*F* = 13.2, *p* < 0.001), NE (*F* = 17.6, *p* < 0.001) and 5-HT (*F* = 13.5, *p* < 0.001) in zebrafish brains decreased by 43%, 50% and 60%, respectively. THY treatment markedly reversed these reductions (Figure 3D–F). These findings indicate that CUS increases stress hormone levels and reduces the secretion of monoamine neurotransmitters, while THY effectively decreases stress hormone levels and modulates neurotransmitter secretion.

### 3.4. THY Regulated the Expression Levels of Inflammatory Factors in the Brains of Zebrafish Following CUS Exposure

**Figure 4:**
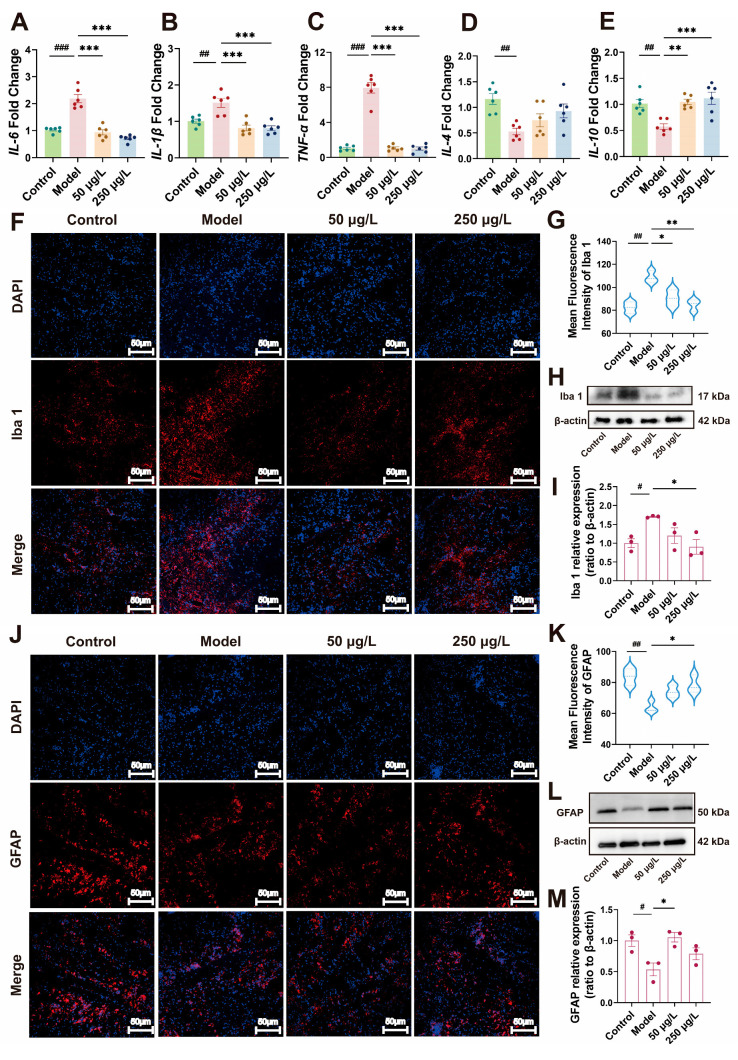
THY reduces CUS-induced inflammation and ameliorates morphological changes in microglia and astrocytes. (**A**–**E**) The mRNA expression levels of IL-6 (*F* = 46.4, *p* < 0.001), IL-1β (*F* = 14.8, *p* < 0.001), TNF-α (*F* = 115.5, *p* < 0.001), IL-4 (*F* = 5.78, *p* = 0.005), and IL-10 (*F* = 9.65, *p* < 0.001) in zebrafish brain tissue were quantified by qRT-PCR (*n* = 6). (**F**) Morphological changes of microglia. Scale bar, 50 µm. (**G**) Quantitative analysis of Iba 1 immunofluorescence staining (*n* = 3) (*F* = 13.2, *p* = 0.002). (**H**) Representative Western blot results of Iba 1 protein expression in brain tissue. (**I**) The quantification of Iba 1 protein Western blots (*n* = 3) (*F* = 5.45, *p* = 0.02). (**J**) Morphological changes of astrocytes. Scale bar, 50 µm. (**K**) Quantitative analysis of GFAP immunofluorescence staining (*n* = 3) (*F* = 8.87, *p* = 0.006). (**L**) Representative Western blot results of GFAP protein expression in brain tissue. (**M**) The quantification of GFAP protein Western blots (*n* = 3) (*F* = 6.53, *p* = 0.02). All data are presented as mean ± SEM. One-way ANOVA was used for overall group comparison. Compared to control group, ^#^ *p* < 0.05, ^##^ *p* < 0.01, ^###^ *p* < 0.001; Compared to model group, * *p* < 0.05, ** *p* < 0.01, *** *p* < 0.001. See Supplementary Figure S4 for the original Western blot image.

The pathogenesis of depression is closely associated with neuroinflammation. An imbalance between pro-inflammatory and anti-inflammatory cytokines contributes to the pathophysiological processes of depression, mainly including tumor necrosis factor-α (TNF-α), interleukin-1β (IL-1β), interleukin-6 (IL-6), interleukin-4 (IL-4), and interleukin-10 (IL-10). To further evaluate neuroinflammatory responses, qRT-PCR was performed to detect the expression of related pro-inflammatory and anti-inflammatory cytokines in the brains of zebrafish. As shown in Figure 4, compared with the Control group, the Model group exhibited significantly upregulated mRNA levels of the pro-inflammatory cytokines IL-6 (*F* = 46.4, *p* < 0.001), IL-1β (*F* = 14.8, *p* < 0.001) and TNF-α (*F* = 115.5, *p* < 0.001), with increases of 1.1-fold, 0.5-fold and 6.7-fold, respectively (Figure 4A–C). Meanwhile, the levels of anti-inflammatory factors IL-10 (*F* = 9.65, *p* < 0.001) and IL-4 (*F* = 5.78, *p* = 0.005) were decreased by 54% and 43%, respectively (Figure 4D,E). Following intervention with THY, the levels of pro-inflammatory cytokines were reduced, whereas anti-inflammatory cytokine expression was restored (IL-4 was not statistically significant). These results indicate that THY effectively modulates the expression of inflammatory cytokines induced by CUS.

### 3.5. THY Effectively Suppresses Excessive Activation of Microglia and Damage to Astrocytes

Microglia and astrocytes are key components of the brain’s innate immune system. Inflammation and stress can trigger abnormal activation of microglia, leading to neuroinflammation and the release of pro-inflammatory cytokines, which further exacerbate neuroinflammatory responses. At the same time, the loss of astrocytes is also a factor in depression. Reduced numbers of astrocytes lead to decreased secretion of neurotrophic factors and weaker protection of neurons, thereby increasing the release of inflammatory factors.

Iba 1 and GFAP are established markers for activated microglia and astrocytes, respectively. Therefore, we performed Iba 1 and GFAP immunofluorescence staining on zebrafish brain tissues. As shown in Figure 4, we observed significant microglial activation in the Model group, with the mean fluorescence intensity increasing by 31.9% (*F* = 13.2, *p* = 0.002) (Figure 4F,G), whereas astrocyte numbers were decreased, accompanied by a 23.5% reduction in mean fluorescence intensity (*F* = 8.87, *p* = 0.006) (Figure 4J,K). Treatment with THY significantly inhibited the abnormal activation of microglia and the damage to astrocytes induced by CUS. Additionally, we assessed the protein expression levels of Iba 1 (*F* = 5.45, *p* = 0.02) (Figure 4H,I) and GFAP (*F* = 6.53, *p* = 0.02) (Figure 4L,M) using Western blotting, which showed consistent trends.

### 3.6. Transcriptomic Studies Reveal an Association Between Depressive-like Behaviors in Zebrafish and the MAPK Signaling Pathway

**Figure 5:**
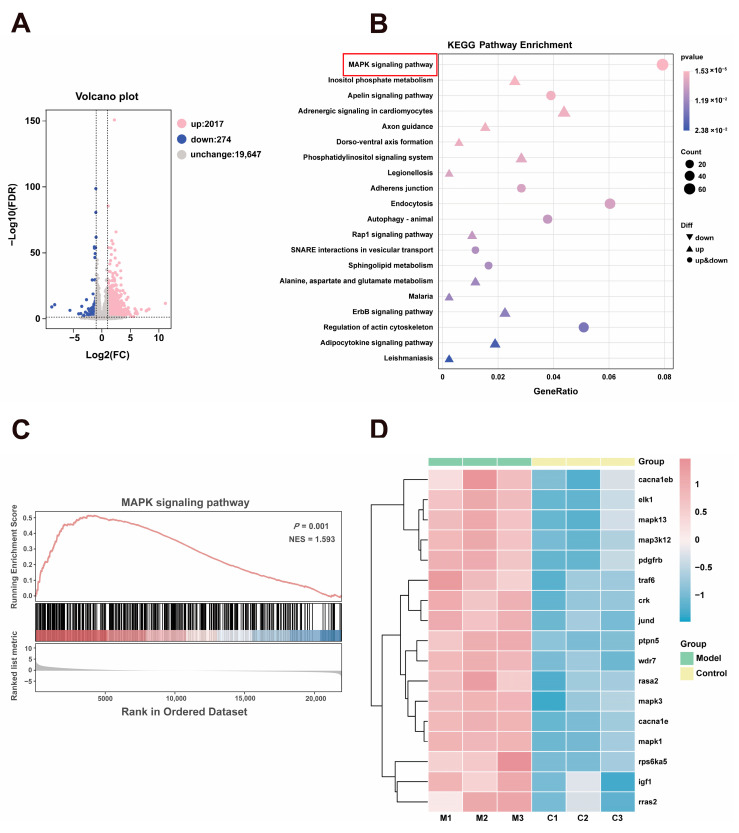
The Transcriptomics of CUS in Zebrafish. (**A**) Volcanic map of differential genes. (**B**) Top 20 most significantly enriched KEGG pathways (The red box indicates the key pathways of our analysis). (**C**) GSEA enrichment map of the MAPK signaling pathway. (**D**) Heatmap of significantly related genes in the MAPK signaling pathway in the transcriptome data that are associated with depression.

To investigate the molecular mechanisms underlying CUS-induced depression-like behaviors, we performed RNA sequencing on brain tissues from Control and Model groups. As shown in Figure 5, analysis revealed 2291 differentially expressed genes compared to the Control group, comprising 274 downregulated and 2017 upregulated genes (Figure 5A). These differentially expressed genes were subsequently subjected to KEGG and GSEA enrichment analyses. KEGG pathway enrichment highlighted the top 20 most significant pathways, among which 10 were found to be associated with depression. These included the ErbB signaling pathway, the MAPK signaling pathway and others (Figure 5B).

The GSEA enrichment analysis showed that MAPK signaling pathway-related genes were significantly enriched at the top of the gene ranking list, with a higher proportion of positively correlated genes (shown in red). This indicates that the MAPK signaling pathway is generally in an activated or up-regulated functional state in the CUS model, demonstrating significant positive enrichment (Figure 5C). Notably, we observed that in the MAPK signaling pathway enrichment results, 17 genes were associated with depression and all showed an upward trend (Figure 5D). Therefore, we hypothesize that the MAPK signaling pathway is one of the key pathways involved in the molecular regulation of depression-like phenotypes in zebrafish. The MAPK signaling pathway participates in regulating neuronal survival, proliferation, differentiation, synaptic plasticity, and inflammatory responses. Chronic stress can lead to dysregulation of MAPK signaling, affecting neurogenesis in brain regions such as the hippocampus and ultimately resulting in neuronal damage.

### 3.7. Network Pharmacology Predicts the Antidepressant Mechanism of THY

**Figure 6:**
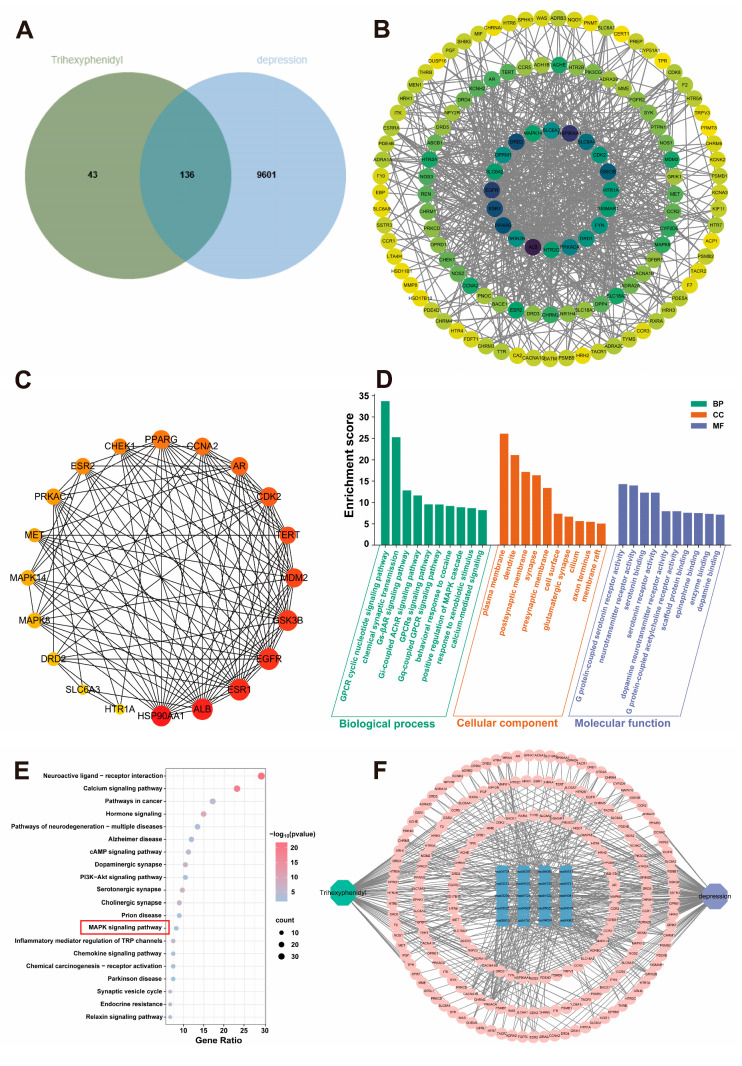
A network pharmacology study investigating THY for the treatment of depression. (**A**) The 136 intersection targets of THY and depression. (**B**) The PPI network of 136 intersection targets. (**C**) The top 20 targets in the PPI network. (**D**) The top 10 BPs, CCs and MFs in GO analysis of 136 intersection targets. (**E**) The top 20 signaling pathways in KEGG enrichment analysis of 136 intersection targets (The red box indicates the key pathways of our analysis). (**F**) The network diagram of “drug–target–pathway–disease”.

Using Swiss Target Prediction and PharmMapper databases, a total of 179 potential targets of THY were identified. As shown in Figure 6, these predicted targets were compared with 9737 depression-related gene targets, resulting in 136 overlapping targets (Figure 6A). A PPI network was constructed and visualized using these 136 overlapping targets (Figure 6B). Subsequently, the cytoHubba plugin was employed to screen the PPI network, and the top 20 hub genes were selected using the maximal clique centrality algorithm. Based on topological analysis, the nodes ranked in descending order of connectivity included key genes such as heat shock protein 90 alpha family class A member 1 (HSP90AA1), albumin (ALB), estrogen receptor 1 (ESR1), and epidermal growth factor receptor (EGFR) (Figure 6C).

GO enrichment analysis of the 136 potential key targets of THY in depression treatment was performed using the DAVID database, covering biological processes (BPs), molecular functions (MFs), and cellular components (CCs). The top 10 results for BPs, MFs, and CCs were ranked by the number of enriched targets and are displayed in Figure 6D, including examples such as the G protein-coupled receptor signaling pathway, neurotransmitter receptor activity, and chemical synaptic transmission. This suggests that THY may exert its therapeutic effects on depression by regulating multiple biological processes. Furthermore, to explore the key pathways involved in the antidepressant action of THY, KEGG pathway analysis was conducted. The top 20 most significantly enriched signaling pathways were identified, including pathways such as neuroactive ligand–receptor interaction, the PI3K-Akt signaling pathway, and the MAPK signaling pathway (Figure 6E). Additionally, Figure 6F illustrates that THY is closely associated with multiple targets and signaling pathways, indicating that its antidepressant effect is likely mediated by multi-target and multi-pathway synergy.

Based on the data provided by transcriptomic analysis and network pharmacology predictions, a systematic comparison and screening were performed, excluding other less relevant pathways. The results showed that the MAPK signaling pathway exhibited the most significant enrichment and strongest association with the key targets. Therefore, the MAPK signaling pathway was selected as the focus for further investigation.

### 3.8. THY Effectively Regulated the MAPK Signaling Pathway in CUS Zebrafish

**Figure 7:**
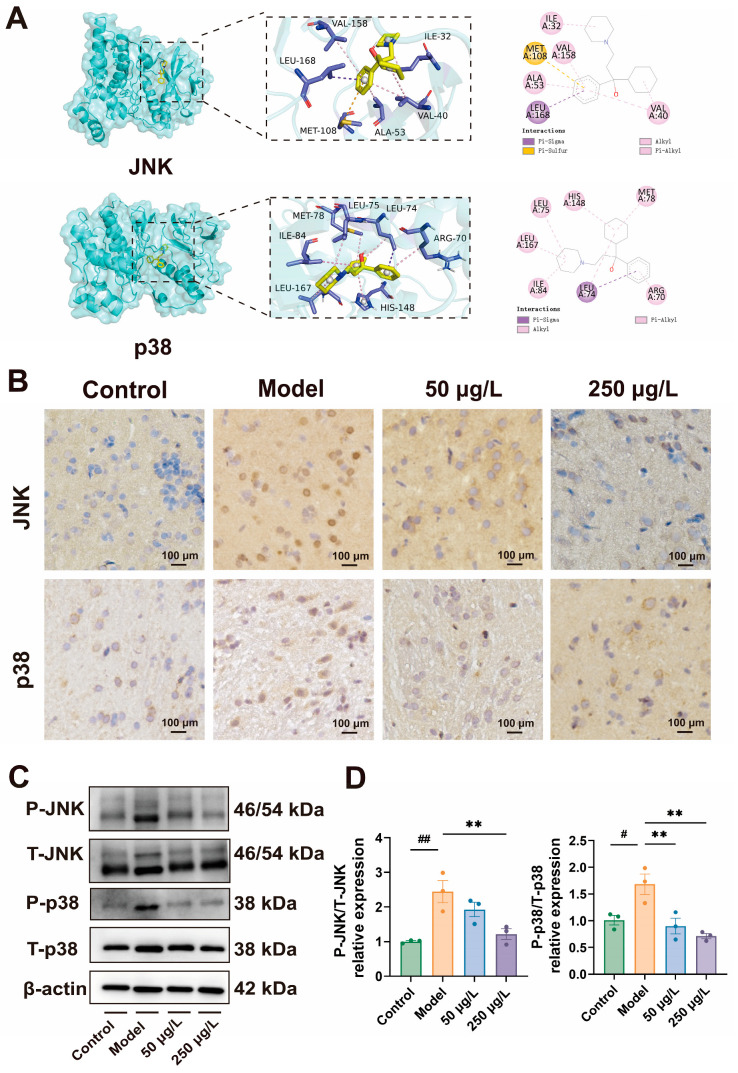
THY regulates the MAPK signaling pathway in the brain of zebrafish induced by CUS. (**A**) Molecular docking results of THY with JNK and p38. (**B**) IHC staining for JNK and p38 in brain tissue. (**C**) Representative Western blots results of P-JNK, T-JNK, P-p38 and T-p38 protein expression in brain tissue. See Supplementary Figure S5 for the original Western blot image. (**D**) The quantification of P-JNK/T-JNK (*F* = 10.2, *p* = 0.004) and P-p38/T-p38 (*F* = 10.4, *p* = 0.004) protein Western blots (*n* = 3). All data are presented as mean ± SEM. One-way ANOVA was used for overall group comparison. Compared to control group, ^#^ *p* < 0.05, ^##^ *p* < 0.01,; Compared to model group, ** *p* < 0.01.

Based on the aforementioned findings, we performed molecular docking studies for JNK and p38 MAPK. As shown in Figure 7, docking predicted that THY could adopt favorable binding poses within JNK and p38, yielding predicted binding free energies (ΔG_bind_) of −7.762 kcal/mol and −8.416 kcal/mol, respectively. These negative ΔG_bind_ values suggest a thermodynamically favorable interaction tendency in the docking model, providing supportive in silico evidence that the JNK/p38 MAPK axis may be involved in the antidepressant-like effects of THY. The docking results between THY and JNK/p38 were visualized using PyMOL 2.3.0 software, and detailed interactions are shown in Figure 7A.

The activation of the MAPK signaling pathway is closely related to neuroinflammation. Persistent activation of the JNK/p38 MAPK pathway has been shown to inhibit neuroplasticity and promote neuronal apoptosis. Therefore, we analyzed the expression of JNK and p38 using immunohistochemistry (Figure 7B) and Western blotting. Western blotting results showed that the levels of JNK (*F* = 10.2, *p* = 0.004) and p38 (*F* = 10.4, *p* = 0.004) were significantly increased in the Model group compared with the Control group. In contrast, treatment with THY markedly reduced the expression of JNK and p38 relative to the Model group (Figure 7C,D). These findings indicate that CUS treatment led to the activation of the JNK/p38 MAPK signaling pathway in zebrafish, while intervention with THY significantly reduced the expression of JNK and p38, effectively modulating JNK/p38 MAPK signaling and thereby attenuating the pathological signaling cascade induced by CUS. These results also confirm the predictions from transcriptomics and network pharmacology.

## 4. Discussion

This study evaluated the antidepressant potential of THY in the CUS model of zebrafish and preliminarily elucidated its underlying mechanism of action. The results showed that THY effectively reversed the neuropathological alterations induced by CUS. In addition, THY attenuated the CUS-induced elevation of stress hormone levels and imbalance of neurotransmitters. Meanwhile, THY alleviated the abnormal activation of microglia and damage to astrocytes, and reduced the aberrant expression of inflammatory cytokines in the brain. Collectively, these improvements ameliorated CUS-triggered anxiety-like and depressive-like behaviors.

The pathogenesis of depression is complex and has not yet been fully elucidated. One of its classic pathological mechanisms is the overactivation of the HPA axis. In patients with depression, the HPA axis is often hyperactive, and elevated levels of CRH, ACTH, and CORT can damage hippocampal neurons, leading to cell death and contributing to anxiety, depression, and other emotional disorders [26]. At the same time, the dysfunction of monoamine neurotransmitter systems (such as DA, NE, and 5-HT) has long been considered the core basis of emotional and motivational regulation disorders [27]. Studies have shown that plasma levels of DA and NE in depressed patients are correlated with symptoms such as psychomotor retardation, anxiety, and low mood [28]. These traditional mechanisms are closely intertwined with the neuroinflammation hypothesis, which has attracted increasing attention in recent years [29]. Chronic stress, a key trigger of depression, can persistently activate immune cells in the central nervous system, primarily microglia and astrocytes [30]. The abnormal activation of microglia releases a large amount of pro-inflammatory cytokines, and excessively high pro-inflammatory factors directly damage neurons and interfere with synaptic function [31]. As key supportive cells responsible for maintaining neuronal homeostasis and the synaptic microenvironment, the impaired function of astrocytes will further worsen the imbalance in neuroinflammation [32]. In this study, we observed that THY treatment effectively reversed HPA axis hyperactivity and neurotransmitter abnormalities induced by chronic stress. It also significantly inhibited microglial overactivation and alleviated astrocyte damage, thereby reducing the expression of pro-inflammatory cytokines. These findings suggest that THY may act simultaneously on multiple pathological pathways in depression, indicating that its antidepressant effect is not achieved through a single mechanism.

Through transcriptomics and network pharmacology analysis, to further investigate the upstream integrative mechanisms, we focused on the MAPK signaling pathway. The JNK and p38 pathways within the MAPK family are primarily involved in biological processes such as inflammatory responses, cell cycle regulation, and cell death, all of which are closely associated with the pathogenesis of depression [33]. Meanwhile, the analysis results indicated that THY exerts its therapeutic effects by regulating multiple targets and pathways. In addition to the MAPK signaling pathway, the core intersecting targets were significantly enriched in depression-related pathways such as the PI3K-Akt and cAMP signaling pathways. Our experimental data indicate that THY specifically inhibits the CUS-induced increase in phosphorylation levels of the JNK/p38 MAPK pathway. This regulatory effect occurs upstream of multiple pathological processes, providing a unified molecular explanation for the downstream observations, including the restoration of neuroendocrine homeostasis, normalization of glial cell function, mitigation of inflammation, and ultimately, neuronal protection and behavioral improvement.

Another strength of this study is the use of the zebrafish model. As an emerging vertebrate model organism, zebrafish offers unique advantages in neuropsychiatric research [34,35,36]. It possesses nearly all major neurotransmitters, hormones, and related receptors, and its neuroendocrine system shares structural and functional similarities with that of mammals [37]. Furthermore, the neural anatomy of zebrafish also exhibits highly conserved features, such as the hippocampus and amygdala [38,39]. These regions have been confirmed to be closely linked to depression-like behaviors. Therefore, by successfully modeling core depressive features in zebrafish using the CUS paradigm, we were able to elucidate the multi-level effects of THY, spanning from molecular pathways and cellular functions to overall behavioral outcomes.

This study also has certain limitations. First, there are still differences between the brain structures of zebrafish and humans, and thus, the findings need to be further validated in more advanced mammalian models. Additionally, each behavioral test in this study was recorded only once per fish. Although single-test recordings are commonly used in zebrafish behavioral research, a single measurement cannot fully reflect the diurnal fluctuations in individual behavior. Future studies may consider repeating tests at multiple time points to enhance the robustness of the results. Second, this study experimentally validated only the MAPK signaling pathway and did not explore the relevance of other pathways to the antidepressant effects of THY in improving depression-like behaviors. Future research should further elucidate its multi-target, multi-pathway mechanisms. There is also a lack of corresponding inhibitors for further exploration. In future studies, relevant inhibitors of the MAPK signaling pathway can be applied to investigate their effects on depressive-like behaviors. Finally, THY is a conventional agent for the treatment of Parkinson’s disease and is currently mainly used to relieve extrapyramidal adverse reactions induced by antipsychotic drugs. The present study aims to explore the ameliorative effects of THY on depressive-like behaviors and its neuroprotective mechanisms, which does not fall within the officially approved research scope of this drug. This study is limited to basic mechanistic research at the animal level and does not involve any clinical medication guidance or medication recommendations.

## 5. Conclusions

Our results indicate that THY can ameliorate the hyperactivity of the HPA axis and the imbalance of neurotransmitters induced by CUS, while regulating the gene expression of inflammatory factors, thereby exerting an antidepressant effect. This process is closely associated with the MAPK signaling pathway. In summary, these findings elucidate the mechanism by which THY improves depression-like behaviors and provide a theoretical basis for the application of THY in the treatment of depression.

## Data Availability

The original contributions presented in this study are included in the article/Supplementary Material. Further inquiries can be directed to the corresponding authors.

## Supplementary Materials

The following supporting information can be downloaded at https://www.mdpi.com/article/10.3390/biom16050678/s1, Figure S1: Depression-like behaviors induced by CUS; Figure S2: Zebrafish acute toxicology experiment; Table S1: Statistical Analysis of Abnormalities in Zebrafish from Acute Toxicity Studies; Figure S3: Morphological assessment of zebrafish; Table S2: The list of primers sequence; Figure S4: Original Western blot image for Figure 4; Figure S5: Original Western blot image for Figure 7C.

## Abbreviations

The following abbreviations are used in this manuscript:

THY	Trihexyphenidyl
CUS	Chronic unpredictable stress
HPA	Hypothalamic–pituitary–adrenal
DA	Dopamine
NE	Norepinephrine
MAPK	Mitogen-activated protein kinase
NTT	Novel tank test
LDB	Light–dark box test
OFT	Open field test
CRH	Corticotropin-releasing hormone
ACTH	Adrenocorticotropic hormone
CORT	Cortisol
5-HT	5-hydroxytryptamine
ELISA	Enzyme-Linked Immunosorbent Assay
Iba 1	Ionized calcium-binding adaptor molecule 1
GFAP	Glial fibrillary acidic protein
GSEA	Gene Set Enrichment Analysis
KEGG	Kyoto Encyclopedia of Genes and Genomes
DAVID	Database for Annotation, Visualization and Integrated Discovery
TNF-α	Tumor necrosis factor-α
IL-1β	Interleukin-1β
IL-6	Interleukin-6
IL-4	Interleukin-4
IL-10	Interleukin-10
BP	Biological processes
MF	Molecular functions
CC	Cellular components
GO	Gene ontology
