# Supplementary material for: Trihexyphenidyl Ameliorates Depression-like Behaviors in Adult Zebrafish Exposed to Chronic Unpredictable Stress, Consistent with Regulation of the MAPK Signaling Pathway
Source: Biomolecules. 2026 May 2;16(5):678. doi: 10.3390/biom16050678 (PMC13204746; doi:10.3390/biom16050678)
Supplement: Supplementary file 1 [file biomolecules-16-00678-s001.zip › biomolecules-4261756-supplementary.pdf]

Supplementary information

Trihexyphenidyl ameliorates depression-like behaviors in adult zebrafish exposed to chronic unpredictable stress, consistent with regulation of the MAPK signaling pathway

Siqi Hu, Yedong Yao, Siyuan Li, Leqing Zhan, Rihua Feng, Dongting Zhangsun, Sulan Luo*, Xiaopeng Zhu*

Guangxi Key Laboratory of Special Biomedicine, School of Medicine, Guangxi University, Nanning, 530004, China

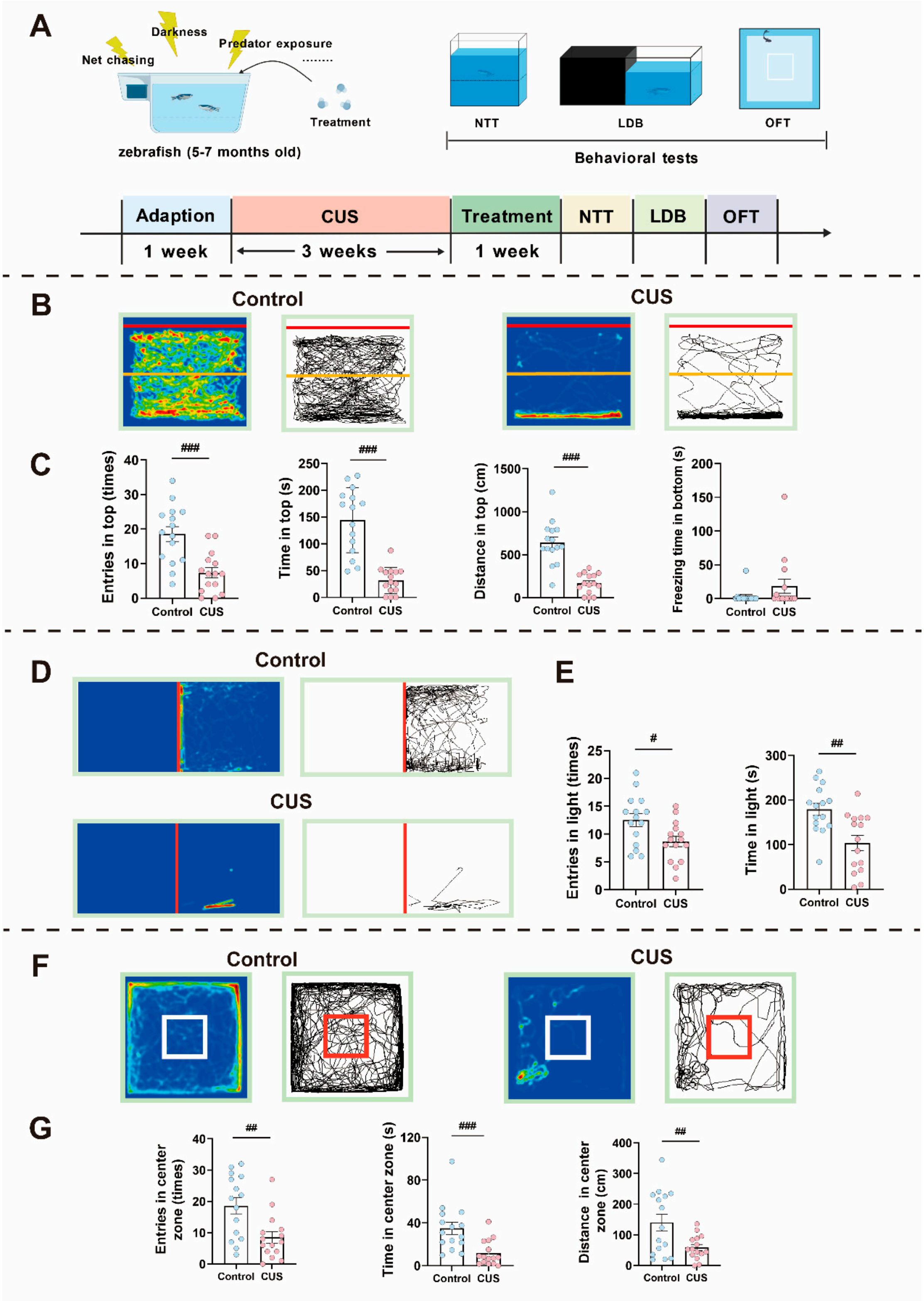

Figure S1. Depression-like behaviors induced by CUS. (A) The flowchart of building the model, created with BioGDP.com. (B) Heatmaps and track plots of zebrafish exploration in the NTT. (C) Entries in top (times) (t (28) = 4.20, $P < 0.001$), time in top (s) (t (28) = 6.67, $P < 0.001$), distance in top (cm) (t (28) =6.642, $P < 0.001$) and freezing time in bottom (s) in the NTT. (D) Heatmaps

and track plots of zebrafish exploration in the LDB. (E) Entries in light (times) (t (28) =2.58, P = 0.02) and Time in light (s) (t (28) =3.47, P = 0.002) in the LDB. (F) Heatmaps and track plots of zebrafish exploration in the OFT. (G) Entries to center zone (times) (t (28) =3.16, P = 0.004), time in center zone (s) (U =31, P < 0.001), and distance in center zone (cm) (t (28) =2.83, P = 0.008) in the OFT. All data are presented as mean ± SEM. Statistical analyses were performed using unpaired two-tailed Student's t-test or two-tailed Mann-Whitney U test. $^{\#}P < 0.05$, $^{\#\#}P < 0.01$, $^{\#\#\#}P < 0.001$.

Table S1

Time \ Concentration (μg/L)	0	50	250	500	750	1000	2000
**24 h**	-	-	-	-	-	-	-
**48 h**	-	-	-	-	-	head up, floating at surface	head up, floating at surface, hypo activity, tail bent downward
**72 h**	-	-	-	floating at surface	head up, floating at surface	head up, floating at surface, hypo activity, tail bent downward	head up, floating at surface, hypo activity, tail bent downward
**96 h**	-	-	-	head up, floating at surface	head up, floating at surface, tail bent downward	head up, floating at surface, hypo activity, tail bent downward	head up, floating at surface, hypo activity, tail bent downward, corkscrew swimming

Statistical Analysis of Abnormalities in Zebrafish from Acute Toxicity Studies

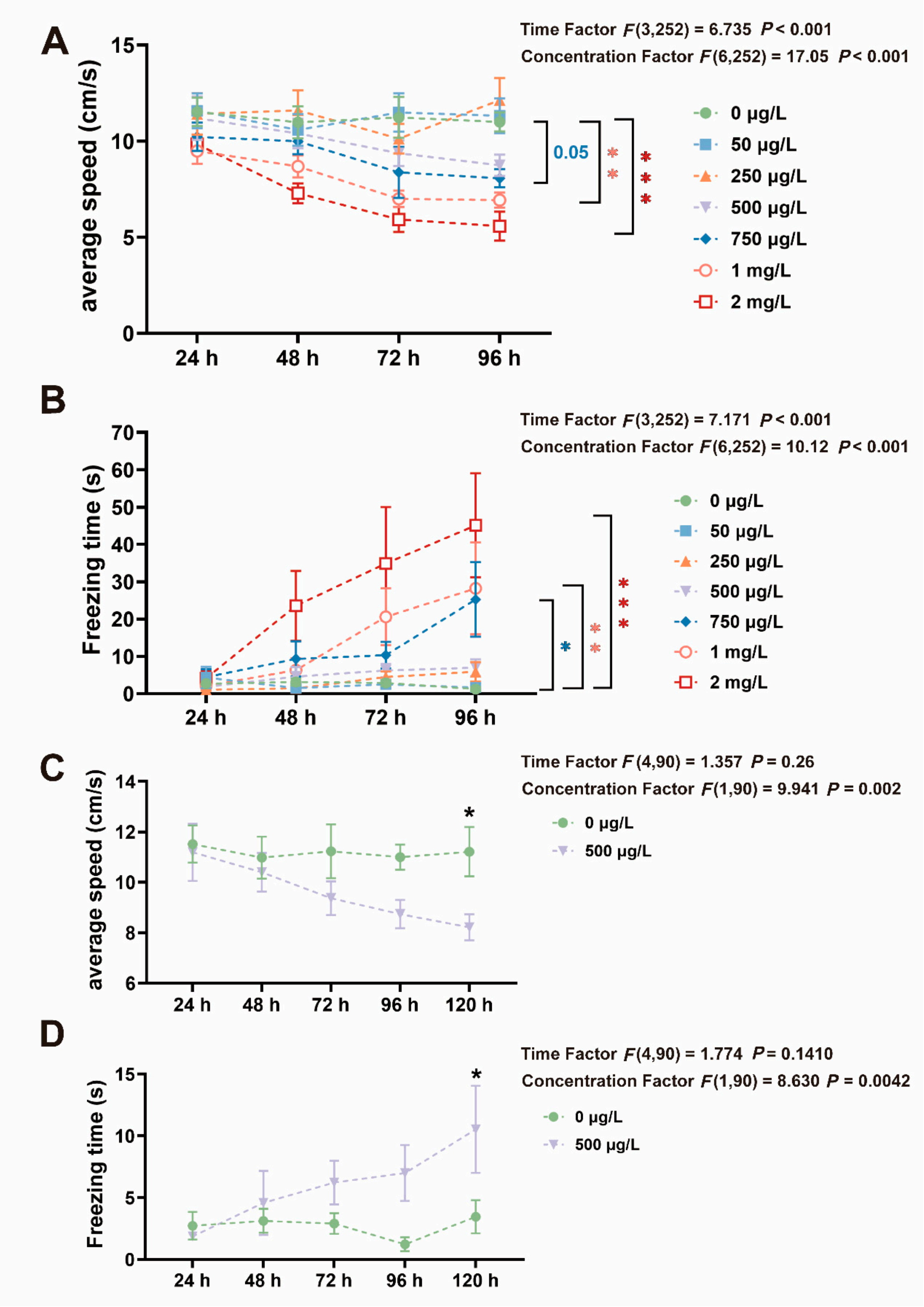

Figure S2. Zebrafish acute toxicology experiment. (A) Average speed (cm/s) within 96 h. (B) Freezing time (s) within 96 h. (C) Average speed (cm/s) at 500 μg/L within 120 h. (D) Freezing time (s) at 500 μg/L within 120 h. All data are presented as mean ± SEM. After verifying the normality and homogeneity of variance of the data, Two-way ANOVA was performed, and Dunnett's test was

used for multiple comparisons to assess differences from the control group. $^{*}P < 0.05$, $^{**}P < 0.01$, $^{***}P < 0.001$.

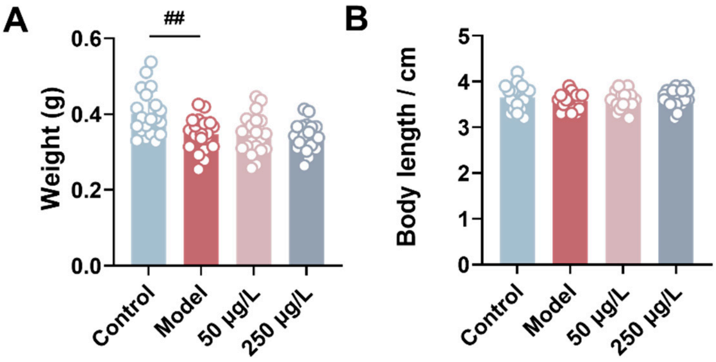

Figure S3. morphological assessment of zebrafish. (A) Zebrafish Weight Statistics. (B) Statistics on Zebrafish Body Length. All data are presented as mean ± SEM. One-way ANOVA was used for overall group comparison. Compared to control group, $^{\#\#}P < 0.01$.

Table S2

Gene	NCBI number	Sequence (5' - 3')	Tm (°C)	Length (bp)[a]
β-actin	NM_131031.2	F: CTGGTCGTTGACAACGGCTCC	60	135
β-actin		R: GGAGTCTTTCTGTCCCATGCC		
IL-6	NM_00126144	F: TCAACTTCTCCAGCGTGATG	55	73
IL-6	9.1	R: TCTTTCCCTCTTTTCCTCCTG		
IL-1β	NM_212844.2	F: CACATCTCGTACTCAAGGAG	55	245
IL-1β		R: CTAGATGCGCACTTTATCCT		
TNF-α	NM_212859.2	F: TCACGCTCCATAAGACCCAG	55	263
TNF-α		R: GATGTGCAAAGACACCTGGC		
IL-4	NM_00117074	F: TTGGTACTTACATTGGTCCC	55	229
IL-4	0.1	R: TCTGTAGATGAGACCTGCTT		
IL-10	NM_00102078	F: TTTTCTCTGGAGTCATCCTTTC	55	103
IL-10	5.2	R: TCCACAAATGAGCAACAGTC		

The list of primers sequence (F: Forward, R: Reverse).

[a] Length refers to the size of the amplified target gene fragment (amplicon) in base pair(bp).

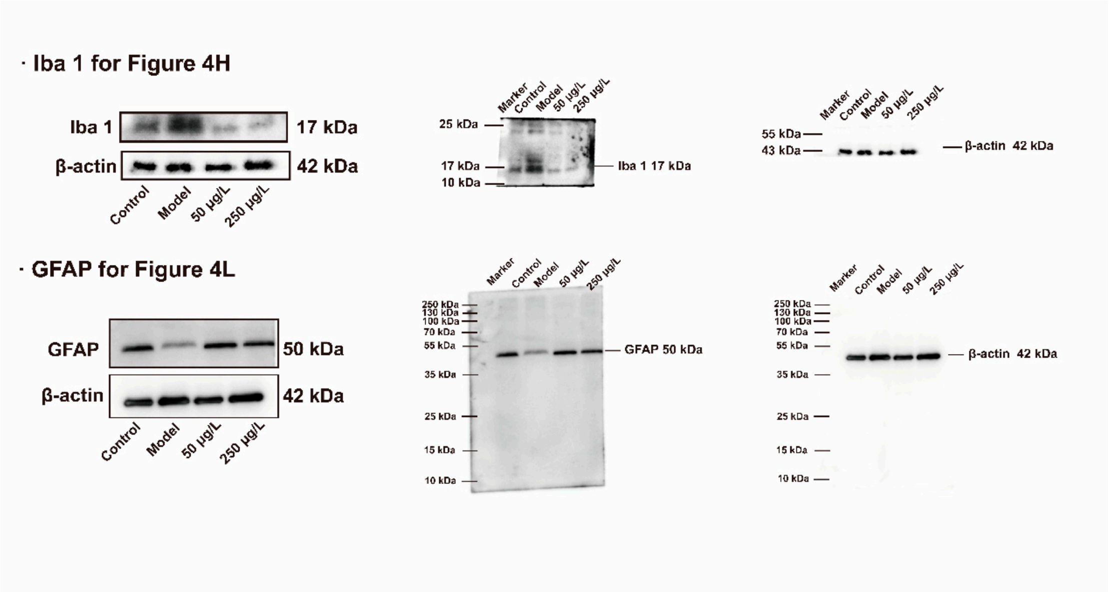

Figure S4. Original Western blot image for Figure 4.

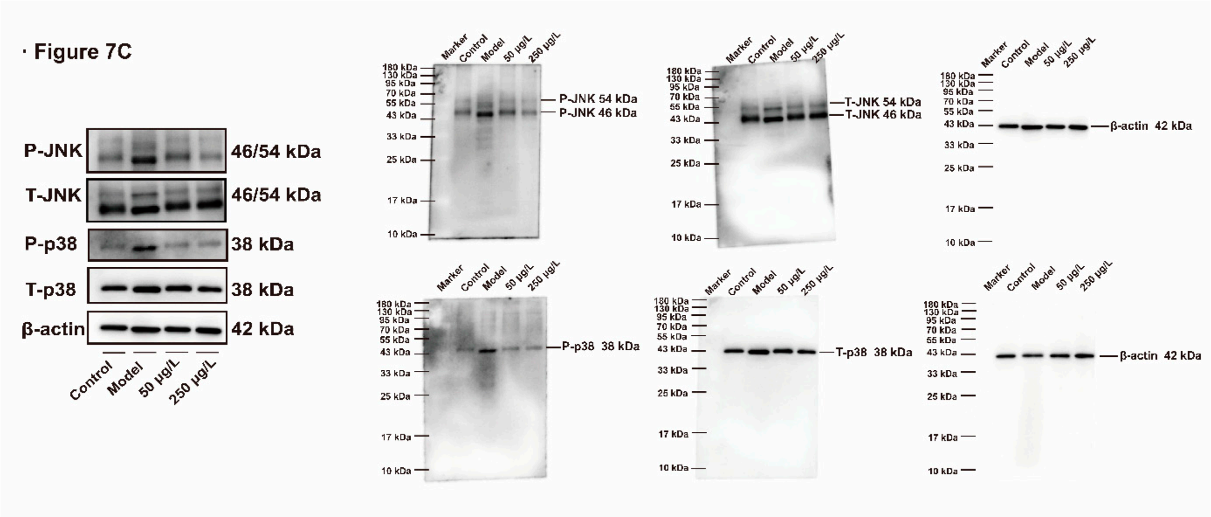

Figure S5. Original Western blot image for Figure 7C.
